# Cardiac Mass and Function Decrease in Bronchiolitis Obliterans Syndrome after Lung Transplantation: Relationship to Physical Activity?

**DOI:** 10.1371/journal.pone.0114001

**Published:** 2014-12-05

**Authors:** Jan B. Hinrichs, Julius Renne, Christian Schoenfeld, Marcel Gutberlet, Axel Haverich, Gregor Warnecke, Tobias Welte, Frank Wacker, Jens Gottlieb, Jens Vogel-Claussen

**Affiliations:** 1 Hanover Medical School, Institute for Diagnostic and Interventional Radiology, Research in Endstage and Obstructive Lung Disease Hannover (BREATH), Member of the German Center for Lung Research, Integrated Research and Treatment Center Transplantation (IFB-Tx), Hannover, Germany; 2 Hannover Medical School, Clinic for Pneumology, Research in Endstage and Obstructive Lung Disease Hannover (BREATH), Member of the German Center for Lung Research, Integrated Research and Treatment Center Transplantation (IFB-Tx), Hannover, Germany; 3 Hannover Medical School, Clinic for Cardiothoracic, Transplant and Vascular Surgery, Research in Endstage and Obstructive Lung Disease Hannover (BREATH), Member of the German Center for Lung Research, Integrated Research and Treatment Center Transplantation (IFB-Tx), Hannover, Germany; University of Pittsburgh, United States of America

## Abstract

**Rationale:**

There is a need to expand knowledge on cardio-pulmonary pathophysiology of bronchiolitis obliterans syndrome (BOS) following lung transplantation (LTx).

**Objectives:**

The purpose of this study was to assess MRI-derived biventricular cardiac mass and function parameters as well as flow hemodynamics in patients with and without BOS after LTx.

**Methods:**

Using 1.5T cardiac MRI, measurements of myocardial structure and function as well as measurements of flow in the main pulmonary artery and ascending aorta were performed in 56 lung transplant patients. The patients were dichotomized into two gender matched groups of comparable age range: one with BOS (BOS stages 1–3) and one without BOS (BOS 0/0p).

**Measurements and Main Results:**

Significantly lower biventricular cardiac mass, right and left ventricular end-diastolic volume, biventricular stroke volume, flow hemodynamics and significant higher heart rate but preserved cardiac output were observed in patients with BOS 1–3 compared to the BOS 0/0p group (p<0.05). In a stepwise logistic regression analysis global cardiac mass (p = 0.046) and days after LTx (p = 0.0001) remained independent parameters to predict BOS. In a second model an indicator for the physical fitness level - walking number of stairs - was added to the logistic regression model. In this second model, time after LTx (p = 0.005) and physical fitness (p = 0.01) remained independent predictors for BOS.

**Conclusion:**

The observed changes in biventricular cardiac mass and function as well as changes in hemodynamic flow parameters in the pulmonary trunk and ascending aorta are likely attributed to the physical fitness level of patients after lung transplantation, which in turn is strongly related to lung function.

## Introduction

Lung transplantation is an established treatment to improve the quality of life and the prognosis of patients with various causes of end-stage lung disease [1]–[3]. The number of lung transplantations increased steadily over the last decade [3]. Bronchiolitis obliterans syndrome (BOS) is one of the main factors of chronic graft dysfunction and a life-threatening complication following initially successful lung transplantation (LTx) in long-term follow up and represents a major limiting factor for long-term survival [3], [4]. Small airway obliteration represents a histomorphological correlate of severe BOS limiting oxygenation, probably altering pulmonary blood flow hemodynamics and pressure, and thus affecting cardiac workload [5], [6]. However, little is known about cardiac function in patients with chronic lung transplant rejection. BOS is characterized by a sustained decline of lung function in spirometric testing after LTx over time [7]. Roughly 50% of the recipients develop BOS five years after transplantation [3]. Over the last years magnetic resonance imaging (MRI) has become a routinely used examination method for the assessment of the lung and heart [8], [9]. Cardiac magnetic resonance imaging (CMR) is a reliable and reproducible tool for studying left and right ventricular volumes and global heart function providing morphological and functional information of the heart and lungs without the use of radiation [9], [10].

This study aimed to determine if there are differences in functional and morphological cardiac MRI-derived parameters and flow hemodynamics in the main pulmonary artery and ascending aorta in a gender-matched cohort with a comparable age range of double lung transplant patients without BOS and with BOS.

## Methods

### Patient Population

**Figure 1:**
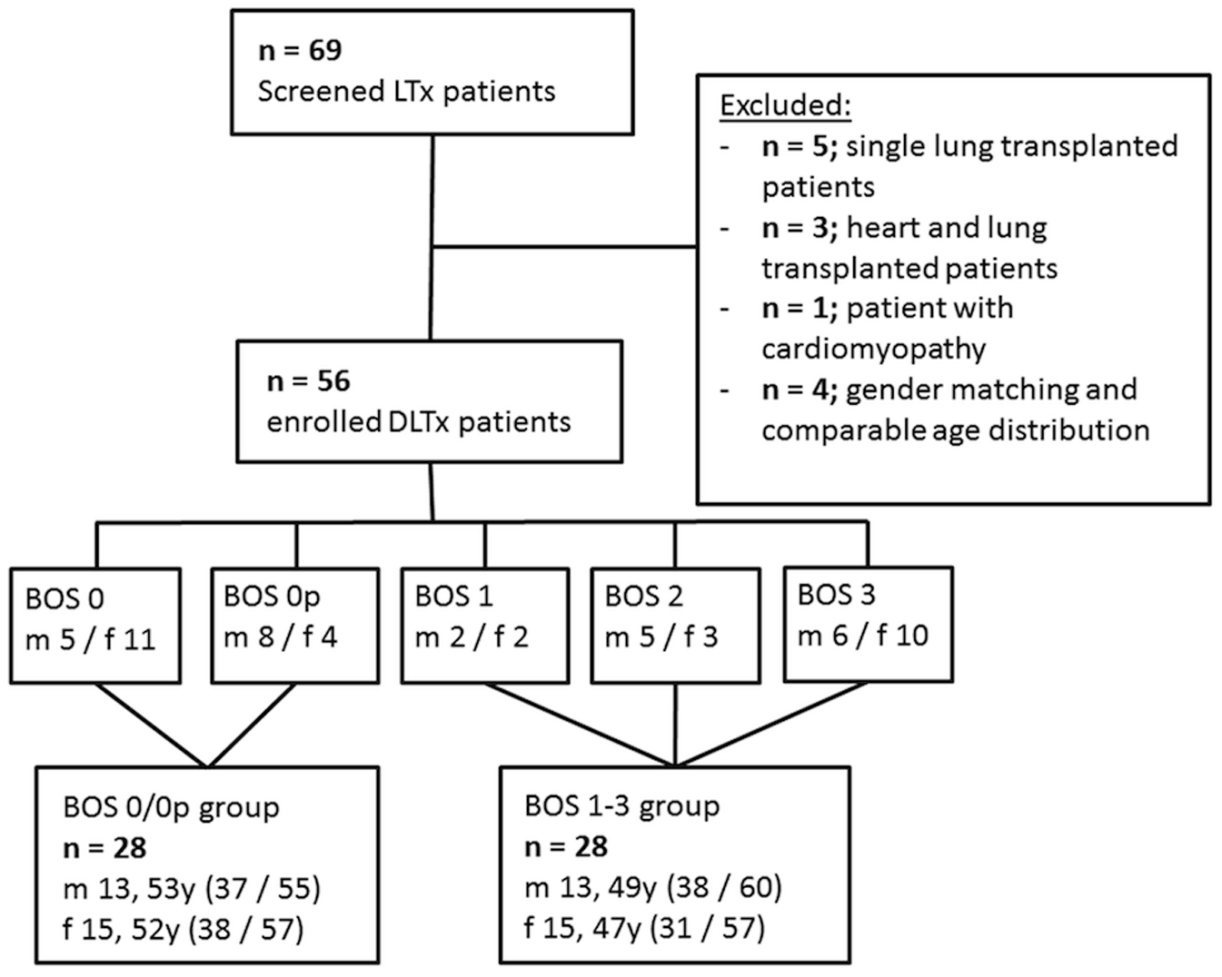
Flowchart of patients included in the study. LTx  =  lung transplantation, DLTx  =  double lung transplantation, BOS  =  bronchiolitis obliterans syndrome, m  =  male, f  = female, y  =  years; median and 25; 75 interquartile range for age distribution.

In this study MRI scans were performed in sixty-nine patients during their regular visit in our lung transplant outpatient clinic between Dec. 2011 and Sept. 2013. Double lung transplanted patients during this period were included in this study. Local ethics committee (ethics commission Hanover Medical School) approval was obtained and all patients gave written informed consent. Exclusion criteria were single lung transplantation, heart and lung transplantation, non-ischemic cardiomyopathy, ischemic cardiomyopathy with left ventricular ejection fraction (LVEF) <50%, and acute pulmonary infections at the time of the MRI scan. Coronary artery disease was present in 6 included patients (4 BOS 1–3), all with an LV EF of ≥50%. Four male patients without BOS (BOS 0) were excluded to achieve gender matching and a comparable age distribution in the two study groups (Figure 1). In our institution the standard immunosuppressive regime consists of cyclosporine A plus mycophenolate mofetil. All immunosuppressive regimes include corticosteroids.

Bronchiolitis obliterans syndrome was diagnosed using lung function test (forced expiratory volume after 1 second (FEV_1_), forced expiratory flow (FEF_25–75_)) as previously described [7]. The spirometric measurements were performed in the Department of Pneumology according to the American Thoracic Society/European Respiratory Society recommendations [11]. BOS stages were dichotomized into two groups: one with BOS (BOS stages 1–3) and one without BOS (BOS 0/0p) (Figure 1) at the time of MRI examination.

Glomerular filtration rate was calculated by use of the chronic kidney disease epidemiology collaboration (CKD-EPI) method as previously described [12]. The clinical diagnosis of hypertension, the duration of hypertension, the use of anti-hypertensive medication and the number of anti-hypertensive drugs was documented. During the outpatient visit of the patients the vital status (e.g. blood pressure) was determined and a routine questionnaire about mobility (walking flight of stairs) was performed. Venous oxygen saturation (SpO_2_) was measured. The indicator for the physical fitness level - the ability of walking stairs - was dichotomized in patients being able to walk 0–1 stairways and patients being able to walk 2 or more stairways for the linear regression model analysis.

### MRI Protocol

All examinations were performed on a commercially available 1.5T MR system (Avanto, Siemens Medical Systems, Erlangen, Germany) equipped with an eight-channel torso phased array coil (Siemens Medical Systems, Erlangen, Germany) in supine position. Retrospectively ECG-gated cine balanced steady-state free precession (bSSFP) sequences were acquired during short inspiratory breath holds in standard short-axis views covering the whole heart using the following parameters: TE  = 1 ms, TR  = 43 ms, flip angle  = 75°, slice thickness  = 8 mm, field of view  = 290×360 mm^2^, matrix size  = 208×256, temporal resolution  = 35 ms, in-plane resolution  = 1.4×1.4 mm^2^, bandwidth/pixel  = 540 Hz/pixel, 30 reconstructed phases. Additionally, phase-contrast flow measurements were performed in the ascending aorta (AO) and the main pulmonary artery (PA) using a through plane retrospectively ECG gated spoiled gradient echo sequence (FLASH) during free breathing. The following acquisition parameters were used: TE  = 2 ms, TR  = 20 ms, flip angle  = 30°, slice thickness  = 5 mm, field of view  = 345×460 mm^2^, matrix size  = 192×256, number of averages  = 3, in-plane resolution  = 1.8×1.8 mm^2^, bandwidth/pixel  = 930 Hz/pixel, 75 reconstructed phases.

### MRI Analysis

Short axis cine MR images and phase-contrast flow measurements were analyzed on a separate workstation with dedicated cardiac software (CVI42 software, Circle Cardiovascular Imaging Inc., Calgary, Canada). Cine images were analyzed by semi-automated contour detection for left and right ventricular endo- and epicardial contours in end-diastole and end-systole by one experienced radiologist. Papillary muscles as well as myocardial trabeculations were included in the blood pool. For calculation of the phase-contrast parameters the same radiologist contoured the border of the AO and the PA on the magnitude images and a semi-automated CVI42 software algorithm completed the contours on the remaining images. Manual corrections were performed if necessary. Using short axis cine images the following parameters were evaluated for the left and the right ventricle: stroke volume (ml), ejection fraction (%), end-diastolic volume (EDV) and end-systolic volume (ESV) (ml), myocardial mass (g) and ventricular mass index (VMI  =  right ventricular (RV) mass/left ventricular (LV) mass) [13]. All values, except for the ejection fraction and VMI, were normalized to body surface area (BSA). Global cardiac mass/BSA (g/m^2^) and cardiac output (l/min) were calculated. Concerning the phase-contrast flow measurements, the following parameters were determined in the AO and the PA: acceleration volume (ml), acceleration time (msec), distensibility (%), maximal systolic flow (ml/sec) and mean systolic velocity (cm/sec) as previously described [14].

### Statistical Analysis

The Shapiro-Wilk test was used to test for normality of distribution for the MRI-parameters and patient population variables. Summary statistical data for outcome and predictor variables were calculated for the BOS 0/0p and BOS 1–3 groups with results expressed as the mean [95% confidence interval] for normally distributed continuous variables, the median (interquartile range) for non-normally distributed continuous variables. Differences in mean values between the two study groups were compared using t-tests and ANOVA between multiple groups. Not normally distributed values were compared using the Wilcoxon test. Differences in proportions for the categorical variables were compared using the Fisher's exact test. Pearson correlation was used to correlate continuous normally distributed parameters. In all tests, a 2-tailed value of 0.05 was defined as the level of statistical significance. Multivariate linear regression was then used to adjust the MRI-derived cardiac function parameters for pertinent demographic and cardiovascular risk factors (systolic and diastolic blood pressure, heart rate, exercise level, time after lung transplantation, use of hypertensive drugs, glomerular filtration rate (GFR), diabetes and age).

Furthermore, a forward-backward stepwise logistic regression analysis (parameters: age, gender, heart rate, GFR, days after LTx, history of hypertension, use of anti-hypertensive medication, history of ischemic heart disease, RV and LV EDV/BSA, ESV/BSA and global cardiac mass/BSA) was performed to analyze if biventricular cardiac mass and MRI-derived function parameters are associated with BOS. In a final logistic regression model (model 1) odds ratios (OR) and respective confidence intervals (CI), as well as p-values were calculated. The detected changes in cardiac mass and function were quite similar to reported changes due to inactivity, thus an additional logistic regression analysis (model 2) was performed with the same parameters from model 1 adding a parameter indicating the physical fitness level (ability of walking stairs) to evaluate the influence of the physical status of the patients to cardiac mass and BOS stadium.

Statistical analysis was performed using commercially available software (JMP 10, SAS Institute, JMP Office Germany, Böblingen, Germany).

## Results

Fifty-six patients, 26 men (52 (interquartiles 40;57) years) and 30 women (49 (35;58) years), participated in this study (p = 0.56 for age; Figure 1). A detailed list of patients' demographics for the two study groups is given in Table 1. Within the BOS group, median time after transplantation was 2101 (1567; 3606) days, differing significantly from the group without BOS, which was 654 (363; 1409) days, respectively (Wilcoxon test, p = 0.0001). The heart rate was significantly higher in BOS patients (t-test, p = 0.007). All 56 participants completed the exams without severe events. Two male patients (BOS 0p 59 y and BOS 3 43 y) did not complete cine MRI due to respiratory distress during the examination. SpO_2_ was significantly different in both groups (p = 0.006), but all patients had normal peripheral oxygen saturation (Table 1). Only two patients in BOS 1–3 group needed oxygen therapy (1–2 L at rest). At the date of the MRI 21 patients (7 with BOS and 14 without BOS) used the standard immunosuppressive regime (cyclosporine A plus mycophenolyte mofetil), 31 patients (18 BOS+; 13 BOS-) changed immunosuppression to tacrolimus plus mycophenolyte mofetil. One patient with BOS used tacrolimus in combination with methotrexate. Two other patients with BOS used tacrolimus either with everolimus or with sirolimus. One patient without BOS used the combination of cyclosporine A plus mycophenolyte mofetil and sirolimus for immunosuppression.

**Table 1:** Patient characteristics.

Characteristics	BOS 1–3 (n = 28)	BOS 0/0p (n = 28)	p value
**Age, years**	48.2 (33.6;57.6)	52.1 (38.3;58.1)	0.99
**Female gender, n (%)**	15 (54)	15 (54)	1 (fisher's*)
**Body surface area, m^2^**	1.73 [1.6;1.8]	1.76 [1.7;1.8]	0.69
**Reason for transplantation**			
Cytic fibrosis, n (%)	10 (36)	8 (29)	
Pulmonary hypertension, n (%)	3 (10)	3 (10)	
emphysema, n (%)	8 (29)	8 (29)	
Pulmonary fibrosis, n (%)	4 (15)	8 (29)	
Other, n	3 (10)	1 (3)	
**Time since BOS diagnosis**	1,111 (517/1,448)	-	
**Time since Tx, days**	2,101 (1,567;3,606)	654 (363;1,409)	0.0001
**Restrictive phenotype, n (%)**	5 (18)	-	
**Blood pressure, mmHg**			
Systolic	134 [129;140]	138 [134;144]	0.32
Diastolic	84 [80;88]	78 [74;82]	0.04
**Diagnosis of hypertension, n (%)**	26 (93)	24 (86)	0.67 (fisher's*)
**Duration of hypertension, years**	5.6 (4.1;8.9)	1.9 (1.2;3.7)	0.0001
**Number of anti-hypertensive drugs, n**	1.9 [1.5;2.3]	1.6 [1.2;1.9]	0.19
**Diagnosis of diabetes, n (%)**	11 (39)	4 (14)	0.07 (fisher's*)
**Glomerular filtration rate, ml/min/1.73 m^2^**	59 [51;68]	69 [57;82]	0.18
**Vital capacity, l**	2.6 [2.4;2.9]	3.4 [3.1;3.8]	0.0003
**FEV_1_ predicted, %**	47 [41;54]	94 [92;97]	0.0001
**venous Oxygen saturation, %**	96.8[95.2;98.5]	98.1[97.0;99.0]	0.006
**Heart rate, beats per minute**	84 [79;88]	74 [69;79]	0.007
**Physical fitness level, stairways to walk**	1.4 [1.0;1.8]	2.5 [2.3;2.8]	0.0001

Median values (25; 75 interquartile range) for not normally distributed values. Mean values [95%-confidence interval] for normally distributed values.

### Cardiac function and BOS

**Figure 2:**
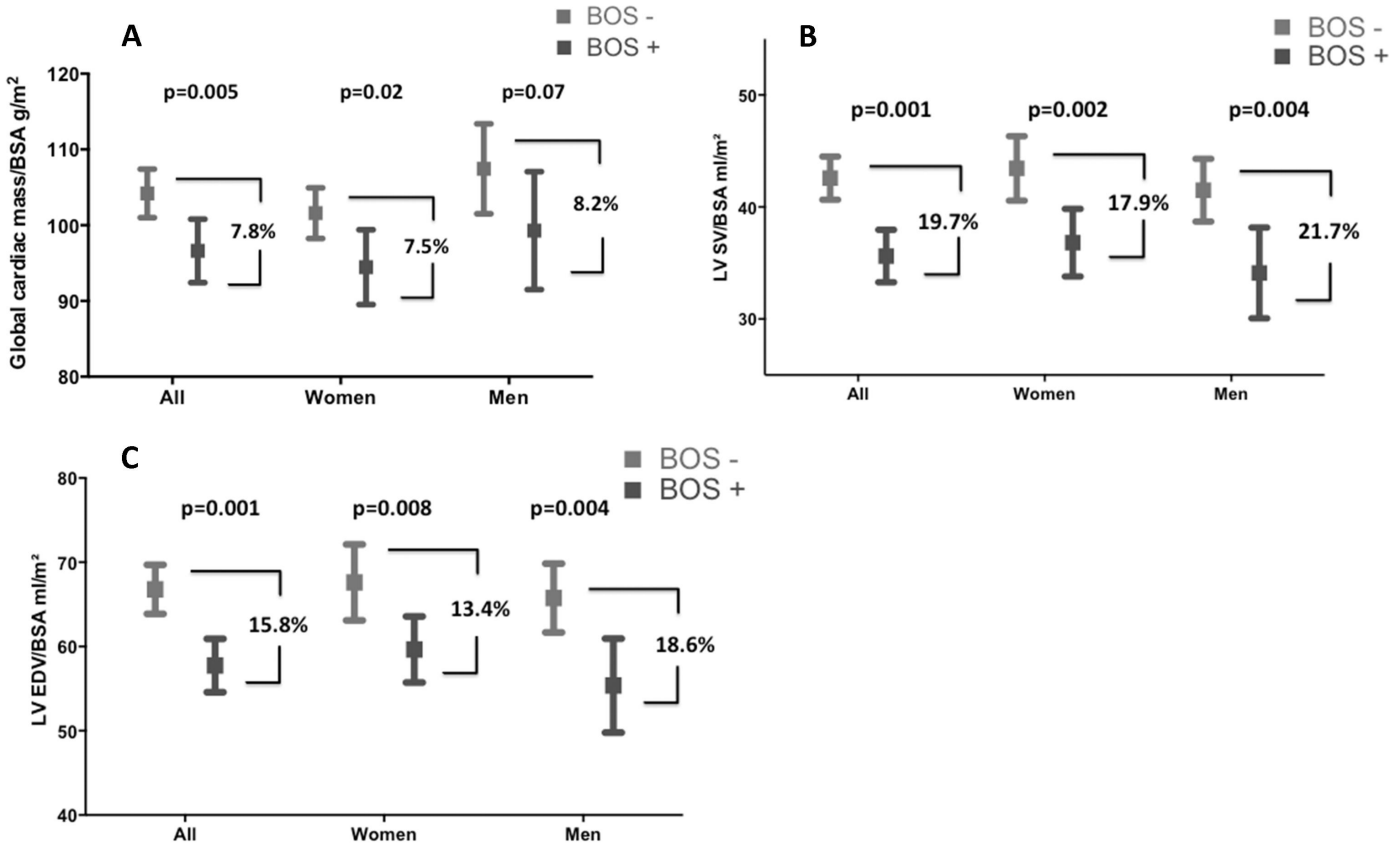
Changes of cardiac parameters. A. Adjusted values of global cardiac mass/BSA (RV + LV mass/BSA) compared BOS 0/0p and BOS1–3 for all patients and for men and women. In all groups a nearly 8% difference in global cardiac mass is seen. **B** Adjusted values of left ventricular stroke volume/BSA compared BOS 0/0p and BOS1–3 for all patients and for men and women. In all groups a 20% difference in stroke volume is seen. **C** Adjusted values of left ventricular end-diastolic volume/BSA compared BOS 0/0p and BOS1–3 for all patients and for men and women. In all groups a significant difference in end-diastolic volume is seen. Shown are mean values and 95%-confidence interval.

Cardiac cine MRI data are shown in Table 2. After adjusting for the above-mentioned pertinent demographic and cardiovascular risk factors RV and LV cardiac mass/BSA, EDV/BSA, EF, SV/BSA and LV ESV/BSA were significantly higher in the BOS 0/0P compared to the BOS 1–3 group (Figure 2a–c and Table 2). Neither in male nor female patients a significant correlation was observed between global cardiac mass/BSA and days after LTx (male: r = −0.27, p = 0.19; female: r = 0.02, p = 0.94). The adjusted ratio of ventricular mass to end-diastolic volume only differed slightly in the LV comparing the two groups (p = 0.02). There were no significant changes in the ventricular mass index between the two groups (p = 0.69).

**Table 2:** Cardiac magnetic resonance imaging (MRI): unadjusted and adjusted values of cardiac structure and function according to bronchiolitis obliterans (BOS) status*.

Cardiac MRI measurements	Unadjusted			Adjusted†			
	BOS 1–3	BOS 0/0p	p	BOS 1–3	BOS 0/0p	% difference‡	p
**Left ventricle (LV)**							
**mass/BSA, g/m^2^**	67 [62;72]	77 [71;83]	0.02	70 [67;72]	75 [73;77]	−7.3	0.003
**end-diastolic volume/BSA, ml/m^2^**	56 [51;62]	67 [60;75]	0.01	58 [55;61]	67 [64;70]	−15.8	<0.001
**end-systolic volume/BSA, ml/m^2^**	22 [19]; [25]	24 [21]; [28]	0.24	22 [21]; [23]	24 [23]; [26]	−9.5	0.014
**stroke volume/BSA, ml/m^2^**	35 [31;38]	43 [39;47]	0.001	36 [33;38]	43 [41;45]	−19.7	<0.001
**ejection fraction, %**	62 [59;65]	647 [63;67]	0.18	62 [60;63]	65 [64;66]	−4.4	0.002
**Right ventricle (RV)**							
**mass/BSA, g/m^2^**	26 [23]; [28]	3 [27]; [33]	0.03	27 [25]; [29]	29 [28]; [31]	−9.2	0.02
**end-diastolic volume/BSA, ml/m^2^**	56 [51;61]	65 [58;71]	0.03	57 [54;60]	64 [62;67]	−13.5	0.002
**end-systolic volume/BSA, ml/m^2^**	23 [20]; [25]	24 [20]; [27]	0.61	23 [22]; [24]	24 [22]; [25]	−2.6	0.47
**stroke volume/BSA, ml/m^2^**	33 [30]; [36]	41 [37;45]	0.002	34 [32]; [36]	41 [39;43]	−21.0	<0.001
**ejection fraction, %**	60 [57;63]	64 [61;67]	0.03	60 [58;61]	64.0 [63;65]	−7.2	<0.001
**Global cardiac mass/BSA, g/m^2^**	93 [87;100]	107 [98;115]	0.013	97 [92;101]	104 [101;107]	−7.8	0.005
**Cardiac output, l/min**	5.0 [4.6;5.4]	5.5 [4.9;6.1]	0.14	5.1 [4.8;5.4]	5.4 [5.2;5.7]	−5.9	0.08

*Values are the mean ± standard deviation [95% confidence interval]. LV  =  left ventricle; RV  =  right ventricle; BSA  =  body surface area; EDV  =  end diastolic volume; ESV  =  end systolic volume; EF  =  ejection fraction; SV  =  stroke volume.

† Adjusted for systolic and diastolic blood pressures, heart rate, exercise level, days after double lung transplantation, use of anti-hypertensive drugs, GFR, diabetes and age.

‡ Percent difference in the adjusted mean values of the indicated variable for the BOS group compared to the group without BOS.

In the described forward-backward stepwise logistic regression analysis, only global cardiac mass/BSA and days after transplantation were significant predictors of BOS (p = 0.046, p = 0.0001, respectively). The results of the respective logistic regression model (model 1) are shown in Table 3. To analyze the possible influence of the physical fitness of the patients the number of stairways they were able to walk was added to the mentioned logistic regression model (model 2; Table 3). In model 2, only days after LTx and physical fitness remained independent predictors for BOS (Table 3). Global cardiac mass/BSA was significantly lower in LTx patients with a lower physical fitness level (t-test, p = 0.02). Also there was a strong direct relationship between the reported fitness level and FEV1% (ANOVA, p<0.0001).

**Table 3:** Logistic regression model analysis for BOS.

Model 1.*	OR	95%-CI	p-value
**Global cardiac mass/BSA, ml/m^2^**	0.96	0.91; 0.99	0.046
**Days after LTx**	1.001	1.0007; 1.002	0.0001

Model 2.†	OR	95%-CI	p-value
**Global cardiac mass/BSA, ml/m^2^**	0.97	0.91; 1.01	0.21
**Days after LTx**	1.001	1.0005; 1.002	0.005
**Physical fitness, (number of stairways)**	0.05	0.002; 0.4	0.01

*Parameters model 1: age, gender, heart rate, GFR, days after LTx, history of hypertension, history of ischemic heart disease, use of anti-hypertensive medication, RV and LV EDV/BSA, ESV/BSA and global cardiac mass/BSA.

† Parameters model 2: parameters from model 1 and physical fitness level.

### Flow Hemodynamics in Aorta and Pulmonary Artery

In the proximal ascending aorta and in the main PA maximal systolic flow, acceleration volume, acceleration time and mean systolic velocity were lower in the BOS 1–3 group compared to the BOS 0/0p group (p<0.05, Table 4). Neither distensibility in the ascending aorta nor in the main pulmonary trunk were significantly different between the two groups (p>0.05). SV/BSA was positively correlated with acceleration volume (PA: r = 0.63, p<0.0001; AO: r = 0.57, p<0.0001), mean systolic velocity (PA: r = 0.24, p = 0.08; AO: r = 0.36, p = 0.008) and maximal systolic flow (PA: r = 0.44, p = 0.001; AO: r = 0.57, p<0.0001). Global cardiac mass was positively correlated with acceleration volume (PA: r = 0.59, p<0.0001; AO: r = 0.58, p<0.0001), mean systolic velocity (AO: r = 0.3, p = 0.03) and maximal systolic flow (PA: r = 0.63, p<0.0001; AO: r = 0.63, p<0.0001). Heart rate was negatively correlated with acceleration volume (PA: r = −0.44, p = 0.0007; AO: r = −0.31, p = 0.02) and acceleration time (PA: r = −0.48, p = 0.0002; AO: r = −0.48, p = 0.0002). After adjusting for systolic and diastolic blood pressure, cardiac output, exercise level, days after double lung transplantation, use of hypertensive drugs, GFR, diabetes and age maximal systolic flow, acceleration volume, acceleration time and mean velocity remained significantly higher in the group without BOS (Table 4).

**Table 4:** Phase-contrast magnetic resonance measurements: unadjusted and adjusted values according to bronchiolitis obliterans (BOS) status*.

Phase-contrast MRI measurements		Unadjusted			Adjusted†			
		BOS 1-3	BOS 0/0p	p	BOS 1–3	BOS 0/0p	% Difference‡	p
**maximal systolic flow, ml/sec**	AO	338 [314;363]	400 [362;437]	0.007	342 [325;358]	386.[371;402]	−13.1	0.002
	PA	313 [286;339]	357 [330;383]	0.02	313 [299;328]	341 [328.3;353]	−8.6	0.005
**acceleration volume, ml**	AO	18 [16]; [20]	23 [21]; [25]	<0.001	18 [17]; [20]	23 [22]; [24]	−23.4	<0.001
	PA	16 [15]; [18]	25 [21]; [28]	<0.001	17 [16]; [19]	23 [22]; [25]	−34.7	<0.001
**acceleration time, msec**	AO	99 [93;105]	109 [101;116]	0.04	99 [95;102]	110 [106;114]	−11.1	<0.001
	PA	102 [94;110]	117 [107;127]	0.02	104 [100;109]	114 [109;120]	−9.7	0.005
**mean systolic velocity, cm/sec**	AO	47 [42;52]	58 [53;64]	0.004	48 [45;52]	57 [53;60]	−17.4	0.008
	PA	46 [42;49]	54 [49;58]	0.002	46 [44;48]	52 [50;54]	−12.8	0.0001

*Values are the mean ± standard deviation [95% confidence interval].

† adjusted for systolic and diastolic blood pressures, heart rate, exercise level, days after double lung transplantation, use of anti-hypertensive drugs, GFR, diabetes and age.

‡ percent difference in the adjusted mean values of the indicated variable for the BOS group compared to the group without BOS.

## Discussion

In this study, a significantly lower biventricular cardiac mass, RV and LV EDV, biventricular stroke volume, a significantly higher heart rate and a preserved cardiac output was observed in patients with BOS 1–3 compared to a gender matched BOS 0/0p group of comparable age range. Similarly, a reduction in flow hemodynamics was observed in the ascending aorta and pulmonary trunk in the BOS 1–3 group. These changes have the footprint of changes due to physical inactivity as previously shown in the community setting in the MESA cohort [15]. The physical fitness level (number of flight of stairs) was significantly lower in the BOS group in our study and strongly associated with global cardiac mass. Therefore, in the second model including physical fitness, global cardiac mass did not remain an independent predictor for BOS.

The 20% difference in biventricular SV mainly resulted from lower biventricular EDVs suggesting a decreased biventricular filling/preload in patients with BOS after lung transplantation likely due to impaired physical fitness in the BOS group. The reverse process of improved ventricular filling with increased LV EDV could be shown in untrained men after short-term endurance training secondary to the Frank-Starling effect [16].

In a non-athletic population of 4,992 ethnically diverse participants free from clinically apparent cardiovascular disease (aged 45–84 years) Turkbey et al. observed that left ventricular mass and end-diastolic volume were positively associated with physical activity with a preserved cardiac output and ejection fraction and with a decreased heart rate [15]. They could show that the relationships were non-linear, with stronger positive associations at lower levels of physical activity as typically seen in patients post lung transplantation. The non-linear relationship observed in the study by Turkbey et al. suggests that a beneficial cardiac response to increased exercise may be possible even in the lowest categories of physical activity and with a lesser response at high levels of exercise [15]. The extent to which any survival benefit from exercise is mediated through myocardial remodeling in patients after lung transplantation is unknown. In their work, they report that the left ventricular mass to volume ratio, a measure of cardiac remodeling, was unchanged over different exercise categories, indicating proportional increases in left ventricular mass and volume with physical activity [15]. Accordingly, the biventricular mass to volume ratio as well as VMI were similar in the BOS 0/0p group and BOS patients in our study.

In recent work from our center, we included 53 patients with advanced lung allograft dysfunction and divided them in two groups according to their exercise capacity [17]. This study showed that low inspiratory capacity and impaired respiratory muscle function were independently associated with decreased exercise capacity. However, survival analysis of time from study inclusion to re-transplantation or death failed to be significantly different between the two groups [17]. In a study by Nathan et al., physical fitness among 42 lung transplant recipients with BOS using the 6 minute walk test as parameter predicted survival and was thought to perform better than spirometry [18].

Phase-contrast flow measurement in the pulmonary trunk and ascending aorta is a well established technique to study flow hemodynamics and flow profiles [14], [19]–[21]. However, little is known about pulmonary or aortic hemodynamics in patients after lung transplantation [22], [23]. Changes in flow hemodynamics in the pulmonary trunk and ascending aorta correlated with biventricular mass and function parameters in our study and are therefore likely also the result of the observed fitness level of our patient cohort. The fact that cardiac mass and function as well as flow hemodynamics are altered in the right heart and pulmonary trunk as well as in the left heart and ascending aorta in the BOS patient group makes a specific effect due to possible altered flow hemodynamics in the pulmonary parenchymal circulation due to BOS less likely.

Airflow obstruction is also present in patients with chronic obstructive airways disease (COPD). In a population-based study, a greater extent of emphysema on CT scanning and more severe airflow obstruction were linearly related to impaired left ventricular filling, reduced stroke volume, and lower cardiac output without changes in ejection fraction after adjusting for covariates except for the level of physical activity in the main analysis [24]. In the same population, extent of emphysema was inversely associated with RV end-diastolic volume, stroke volume, and mass after adjusting for several covariates except for the level of physical activity [25]. If the observed changes in cardiac mass and function as well as flow hemodynamics in our patient cohort after lung transplantation are due to airflow obstruction/hyperinflation in addition to the changes attributed to the physical fitness level needs to be determined in future studies. Notably, a direct relationship of FEV_1_% and fitness level was present in our patient cohort as well.

All of the patients had a time since transplantation of at least 6 month. Kasimir et al. perioperatively investigated changes in right and left ventricular parameters by echocardiography in a group of single lung transplanted patients with pulmonary hypertension early after transplantation [26]. During a time period of 3 months a reverse cardiac remodeling was shown and normal values were reached [26]. Therefore it is unlikely that cardiac mass and function parameters in the presented study were influenced by pre-transplantation lung disease. One major difficulty comparing the two investigated BOS groups is the significant difference in time after transplantation. Thus time after transplantation was included as parameter in the logistic regression models together with the MRI derived parameters for prediction of BOS. Longitudinal MRI data would be a possibility to study intra-individual changes over time in patients after lung transplantation.

We observed a difference of about 8% in biventricular cardiac mass for male and female patients with a higher cardiac mass in patients without BOS. Cardiac mass is known to increase with the level of exercise as shown in several studies [15], [27], [28]. It is also known that a prolonged bed rest (6 to 12 weeks) leads to an atrophy of the heart of at least 10% to 15% of LV mass [29]. In the study of Turkbey et al., the highest level of intentional exercises comes along with an increase of the mean LV mass/BSA up to 3.1 g/m^2^ [15]. In our study, the potential influence of a reduced physical fitness was evaluated with a semiquantitative parameter (walking number of stairs). A longitudinal study with an integrated standardized fitness parameter (e.g. 6 minute walk test or a pedometer) could evaluate the cardiac changes related to physical fitness in patients after LTx, its association with BOS degree and possible effects on patient survival.

Also age and gender are important factors influencing cardiac mass and function [27]. Physiological ageing is known to be associated with a smaller heart size. Sandstede et al. showed in healthy volunteers that a lower LV mass is associated with female sex and older age [27], [30]. For this reason, our study cohort was sex-matched with comparable age distribution in the two study groups.

Patients with BOS had a significantly lower SpO_2_ values compared to patients without BOS. Overall, all patients had a normal peripheral SpO_2_, thus a theoretically possible hypoxia-related pulmonary arterial hypertension seems unlikely in our cohort.

Calcineurin inhibitors are known to effect cardiac hypertrophy in mice positively [31]. Also, mammalian target of rapamycin inhibitors (mTOR) are reported to lower cardiac mass in heart or kidney transplant recipients [32]. Due to the fact, that all lung transplant recipients were treated with either cyclosporine A or tacrolimus and that only 3 of the patients in the study had an immunosuppressive regime including a mTOR inhibitor (Sirolimus or Everolimus; 2 patients with BOS, 1 patient without BOS) we do not expect any influence on cardiac mass due to the immunosuppression regime in the presented study.

Left ventricular mass is a well-known predictor for incident cardiovascular events e.g. heart failure, sudden death and cardiovascular mortality representing a minor factor of death after LTx (about 5%) [3], [33]–[35]. Also, hypertension and treatment of hypertension with anti-hypertensive medication are known to influence cardiac remodeling [36], [37]. In our study there was no significant difference between the use and number of anti-hypertensive drugs or the diagnosis of hypertension between the two examined BOS groups. Also, the influence of the renin-angiotensin-system on cardiac mass is known (35). The GFR serves as a parameter for the renin-angiotensin-system and was not significantly different between the two investigated groups of lung transplant patients. A limitation of our study is the adjustment of the MR parameters to only one systolic and diastolic value instead of repeated measurements in a controlled setting [15].

We conclude that the observed changes in biventricular cardiac mass and function as well as changes in hemodynamic flow parameters in the pulmonary trunk and ascending aorta are likely attributed to the physical fitness level of patients after double lung transplantation, which in turn is strongly related to lung function.
