# Correction: Cardiac Mass and Function Decrease in Bronchiolitis Obliterans Syndrome after Lung Transplantation: Relationship to Physical Activity?

**DOI:** 10.1371/journal.pone.0119999

**Published:** 2015-03-19

**Authors:** The PLOS ONE Staff

There is an error in Table 2. The value for unadjusted Right ventricle (RV) mass/BAS, gm/m2 in the BOS 0/0p is incorrect. The correct value is 30. Additionally, the value for the unadjusted ejection fraction, % BOS 0/0p is incorrect. The correct value is 64. Please see the corrected Table 2 here. The publisher apologizes for the error.

**Table 2:** Cardiac magnetic resonance imaging (MRI): unadjusted and adjusted values of cardiac structure and function according to bronchiolitis obliterans (BOS) status*.

Cardiac MRI measurements	Unadjusted			Adjusted †			
	BOS 1–3	BOS 0/0p	p	BOS 1–3	BOS 0/0p	% difference ‡	p
Left ventricle (LV)							
**mass / BSA, g/m** ^**2**^	67[62;72]	77 [71;83]	0.02	70 [67;72]	75 [73;77]	−7.3	0.003
**end-diastolic volume / BSA, ml/m** ^**2**^	56 [51;62]	67 [60;75]	0.01	58 [55;61]	67 [64;70]	−15.8	<0.001
**end-systolic volume / BSA, ml/m** ^**2**^	22 [19;25]	24 [21;28]	0.24	22 [21;23]	24 [23;26]	−9.5	0.014
**stroke volume /BSA, ml/m** ^**2**^	35 [31;38]	43 [39;47]	0.001	36 [33;38]	43 [41;45]	−19.7	<0.001
**ejection fraction, %**	62 [59;65]	65 [63;67]	0.18	62 [60;63]	65 [64;66]	−4.4	0.002
**Right ventricle (RV)**							
**mass / BSA, g/m** ^**2**^	26 [23;28]	30 [27;33]	0.03	27 [25;29]	29 [28;31]	−9.2	0.02
**end-diastolic volume / BSA, ml/m** ^**2**^	56 [51;61]	65 [58;71]	0.03	57 [54;60]	64 [62;67]	−13.5	0.002
**end-systolic volume / BSA, ml/m** ^**2**^	23 [20;25]	24 [20;27]	0.61	23 [22;24]	24 [22;25]	−2.6	0.47
**stroke volume /BSA, ml/m** ^**2**^	33 [30;36]	41 [37;45]	0.002	34 [32;36]	41 [39;43]	−21.0	<0.001
**ejection fraction, %**	60 [57;63]	64 [61;67]	0.03	60 [58;61]	64 [63;65]	−7.2	<0.001
**Global cardiac mass / BSA, g/m** ^**2**^	93 [87;100]	107 [98;115]	0.013	97 [92;101]	104 [101;107]	−7.8	0.005
**Cardiac output, l/min**	5.0 [4.6;5.4]	5.5 [4.9;6.1]	0.14	5.1 [4.8;5.4]	5.4 [5.2;5.7]	−5.9	0.08

* Values are the mean ± standard deviation [95% confidence interval]. LV = left ventricle; RV = right ventricle; BSA = body surface area; EDV = end diastolic volume; ESV = end systolic volume; EF = ejection fraction; SV = stroke volume.

† Adjusted for systolic and diastolic blood pressures, heart rate, exercise level, days after double lung transplantation, use of anti-hypertensive drugs, GFR, diabetes and age.

‡ Percent difference in the adjusted mean values of the indicated variable for the BOS group compared to the group without BOS.
